# Low-Level Knowledge and Associated Factor of Sexual and Reproductive Health Rights Among Aksum University Students, Aksum Ethiopia

**DOI:** 10.3389/fpubh.2022.860650

**Published:** 2022-05-20

**Authors:** Elsa Tesfa Berhe, Mlite Ayene, Tsigehana Gebregyorgis, Merhawit Gebremeskel Hagos, Teferi Gebru Gebremeskel

**Affiliations:** ^1^Department of Reproductive Health, College of Health Sciences, Aksum University, Aksum, Ethiopia; ^2^Department of Epidemiology and Biostatistics, College of Health Sciences, Aksum University, Aksum, Ethiopia; ^3^Department of Nutrition, College of Health Sciences, Aksum University, Aksum, Ethiopia; ^4^Department of Midwifery, College of Health Sciences, Aksum University, Aksum, Ethiopia; ^5^Discipline of Public Health, Flinders University, Adelaide, SA, Australia

**Keywords:** knowledge, sexual and reproductive rights, University students, Ethiopia, associated factors

## Abstract

**Background:**

Reproductive health rights in Africa are unknown. According to international studies, reproductive health rights of young people particularly university students, are violated, and most of these violations go unreported due to a lack of information and awareness of these rights. The aim of this study was to assess the level of knowledge and associated factors on sexual and reproductive health rights in Ethiopia.

**Methods:**

Institution-based cross-sectional study using an interviewer-administered and structured questionnaire was conducted. The study population consisted of students of the selected department between November, 2018 and June, 2019. A simple random sampling method technique was used to select 420 students. Bi-variate and multi-variate logistic regression analysis was performed.

**Results:**

The knowledge level of the respondents was 16.4%. The majority of students 205 (51%) were in the age group < 20 years. Male [*AOR*: 1.46, 95% *CI*: 1.09–1.95]; coming from urban areas [*AOR*: 2.11, 95% *CI*: (1.02–4.37)]; provision of sexuality education or lecture in departments [*AOR*: 1.39, 95% *CI*: 1.02–1.91] and discussion about reproductive health with anyone else [*AOR*: 2.31, 95% *CI*: 1.48–3.62] were significant association for the knowledge of sexual and reproductive health rights.

**Conclusions:**

Respondents' knowledge level was found to be very low. Therefore, strengthening students' lectures or education on reproductive health in the curricula of high school, encouraging discussions with parents, and anyone might improve the knowledge toward sexual and reproductive health rights.

## Introduction

More than 1.5 billion young people and adults between the ages of 10 and 24 years live in the About 78% of these people live in poor areas of Asia and Africa (1). It is the second leading cause of disease, disability, or death in developing countries with effective interventions to address the problem (2). Lack of knowledge about reproductive biology and health is critical to their ability to protect theme selves from unwanted reproductive disorders. Although there is not enough knowledge to prevent such an outcome, it is the right and desire of all adults to have this information (3).

According to an international study, 7–48% of adolescent girls and 0.2–32% of adolescents boys, have had their first sexual intercourse was forced (4). The growing number of sexual and reproductive rights violations have been highlighted in the media, with a few cases appearing in court (5). Sexual assault was reported in 3–7% of men in 19 countries. Every 5 min, a young man commits suicide because of sexual and reproductive health problems (6).

Level of knowledge of sexual and reproductive health rights among University students was in Ghana (80%) (7), Ikenne (63.2%) (8), Ikorodu (62.3%) and Ikeja (60.3%) (3), and Ethiopia, the level knowledge varied between 31.6 and 67%. In Adet Tana Haik college students (59.6%) (9), Gondar University (57.7%) (10), Wolaita Sodo, Ethiopia (54.5%) (2) Southwest Nigeria (3). In East Gojjam, Ethiopia (67%) (11) but Harar (31.6%) (12) and Shire town (47.1%) (13). According to various kinds of literature, the reason why it's associated with the social display and knowledge of sexual and reproductive health rights; age, location, elementary and secondary school, faculty, field study, parental education, work, and discussion. Sexual reproductive features of health services such as the use of RH services, resources, and participation in RH clubs, sexual reproductive health issues, early sexual education, or discourse (14–24).

Sexual and reproductive health and reproductive rights issues are currently featured on the SDG agenda, but opportunities exist to expand their presence at both the global and national levels, by establishing sexual and reproductive health and rights (SRHR)–specific indicators to measure progress toward the SDGs (25).

SRHRs are a prerequisite for achieving SDGs and including gender-related goals in health education (25), however, thousands of development goals have been unsuccessful in SRHR issues. Therefore, the purpose of this quantitative evidence synthesis (QES) is to develop the concept of SRHR issues from responsible bodies. The findings provide the appropriate support to improve knowledge level based on realistic recommendations made by the WHO.

## Methods

The study was conducted at Aksum University, which is located in the city of Aksum, Tigray Region (North Ethiopia). Aksum has located at 1,024 km Northwest of Addis Ababa and 241 km far away from Mekele. It was established in 1999 E.C as the university. There are three campuses. Currently, the university is operating with 10 colleges and/or faculties comprised of a total of 60 departments. There was a Gender and anti-HIV/AIDS club operating at the University. According to the registrar's office of the university report, in 2011 E.C there were about 15,000 students in the university and about 13,000 were regular undergraduate students, and the study was conducted from November 2018 to June 2019 E.C.

The institution-based cross-sectional study was conducted among regular undergraduate students to assess the level of knowledge and associated factors on SRHRs. The study period was from November 2018 to June 2019. Data collection was carried out from 1st to 30th March, 2019. Students of selected departments during the study period were taken because of the study populations. All regular undergraduate students during the study period were included, where as students who were seriously ill and unable to respond to the questions were excluded from the study.

The sample size was 420 determined using a formula comparison of proportions. The internal comparison was done using a Discussion about Rh with anyone as an associated factor for the level of knowledge of SRHR. Following sample size determination first, students were adjusted based on the first, second, and third year of study and above. From 10 collages, students in each category were selected using a simple random sampling method. The number of students per sample was proportional to the total number of students. Knowledge about SRHRs, the dependent variable, was measured by using 24 questions and each question contains “0 = No” and “1 = Yes” alternatives. As a result, the total score was (0–24) and participants who scored above the mean score were considered as knowledgeable. Sexual Experience: sexual contact or intercourse at least once in the past. Data were collected using an interviewer-administered and check lists. To establish face validity and translation quality, the questionnaire was tested on 5% of the total sample size outside of the study site by data collectors and supervisors. The data were collected by the health care provider.

Data were coded, cleaned, recorded, and entered Epi-Info window version 7 statistical programs were used to clean and enter the data into a computer. For further data, exporting and analysis SPSS windows version 22.0 was used. Knowledge of SRH use between each predictor and predicted variable was initially performed by bivariate analysis. Each predictors' odds ratios (*OR*) at 95% confidence intervals (*CI*) and *p*-values were calculated. Then all statistically significant variables at *p* < 0.05 were entered into multivariate analysis using the logistic regression model. Finally, the results were presented in the form of tables, and summary statistics.

Ethical clearance was obtained from the Institutional Review Committee (IRC), College of Medicine and Health Sciences, University of Aksum. Permission letter was received from those faculties and departments' verbal and written consent was obtained after explaining their full right to refuse, withdraw any time, without any explaining or giving reasons. Information's obtained from individuals' participants was kept secure and confidential. Names and other identifying data of respondents were made by using code throughout the study process to obtain confidentiality. Finally, data were collected according to the standard questionnaire prepared.

## Results

### Socio-Demographic Characteristics

A total 420 respondents were included in the prospective analysis. The mean age was 21.3 ± 1.7 years. The minimum and maximum ages were 18 and 26 years, respectively. The majority of students (51%) were in the age group < 20 years. The majority of the students 83.6% students were single, 8.7% married, and 5% divorced. More than half of the students were female (58.5%), and 37.6% were the first-year student (Table 1).

**Table 1:** Socio-demographic, parent's education and occupation, Sexual experience, and RH service utilization of participants in the study of knowledge on SRHR at Aksum University, northern Ethiopia, 2018/2019.

**Variable**		**Frequency**	**Percentage (%)**
Age	<20	205	51
	20–24	176	43.8
	≥25	21	5.2
Sex	Female	235	58.5
	Male	167	41.5
Religion	Orthodox	339	84.3
	Muslim	19	4.7
	Protestant	44	10.9
Marital status	Unmarred	347	83.6
	Married	35	8.7
	Divorced	20	5
Residence	Rural	194	48.3
	Urban	208	51.7
Ethnicity	Amhara	200	49.8
	Oromo	63	15.7
	Tigrigna	94	23.4
	South	38	9.5
	Others	7	1.6
Type of school attended in elementary and secondary	Public	317	78.9
	Private	55	13.7
	Both	30	7.5
Department	History	18	1.5
	Computer science	31	3.8
	Animal science	50	15.7
	Horticulture	48	15.5
	Geography	30	3.6
	Journalism	43	13.2
	Law	38	9.8
	Midwife	20	2.6
	Pharmacy	25	2.9
	Public health	47	15.4
	Medical laboratory	52	16
Year of study	First-year	151	37.6
	Second-year	150	37.3
	Third-year and above	101	25.1
Father educational status	Unable to read and write	141	35.6
	able to read and write	110	27.4
	Elementary	58	14.4
	Secondary	13	32
	College and above	80	19.9
Fathers occupation	Governmental employee	75	18.7
	Private employee	12	3
	Merchant	131	32.6
	Farmer	179	44.5
	Others	5	1.2
Mothers educational status	Unable to read and	202	50.2
	Write	122	30.3
	Able to read and write	41	10.2
	Elementary	22	5.5
	Secondary	15	3.7
	College and above		
Mothers occupation	Housewife	260	64.7
	Governmental employee	53	13.2
	Merchant	38	9.5
	Farmer	41	12.4
	others	9	1.1
Have you ever had sex	Yes	114	28.6
	No	288	71.4
Age at first sex	<18	35	19.2
	18–20	52	66
	>20	27	14.8
When to start sex	Before joining university	69	54.8
	After joining university	45	45.2
	Total	114	100
No of the sexual partner	One	46	42.6
	Two	16	12.6
	Three and above	52	44.8
	Total	114	100
With who had you discussed sexual issues	Mother	33	18.6
	Father	4	1
	Sister	30	12.5
	Peers	119	36.5
	Schoolteacher	26	10.9
	Health professional	73	20.5
	Total	285	100

### Parents Educational and Occupational Status, Sexual Experience and RH Service Utilization

About one-quarter (27.4%) of the respondents can read and write but have no formal education, while 44.5% are farmers. Nearly half of the participants 202 (50.2%) had no formal education but 15 (3.7%) completed secondary education. Of all respondents, 114 (28.6%) had sexes and the first age of first sexual contact was 18.5 (SD ± 2.1) years. About two-thirds (66%) are between the age of 18–20 years and 45.2% being after university. Of this, 52 (44.8) have many sexual partners in their lives (Table 1).

**Figure 1:**
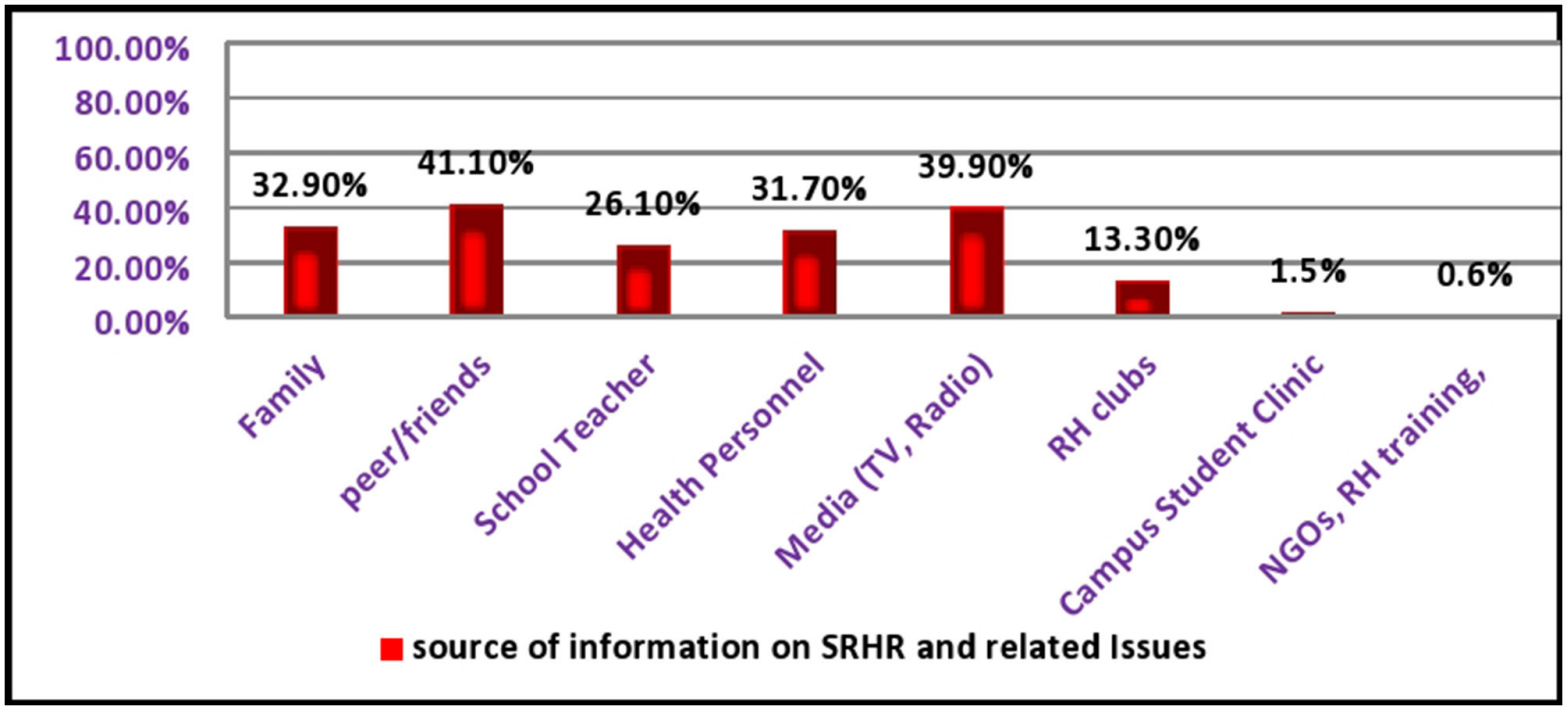
Source of information on SRH and related issues.

More than one-fifth (22.2%) participate in reproductive health clubs and about 193 (48%) of campus students in the university provide RH services, and 33.8% don't know which services they use. More than two-fifth (41.1%) use their friends as a source of information on SRH issues, and only 12 (1.5%) use campus student clinics (Figure 1).

### Knowledge of Sexual and Reproductive Health Right

**Figure 2:**
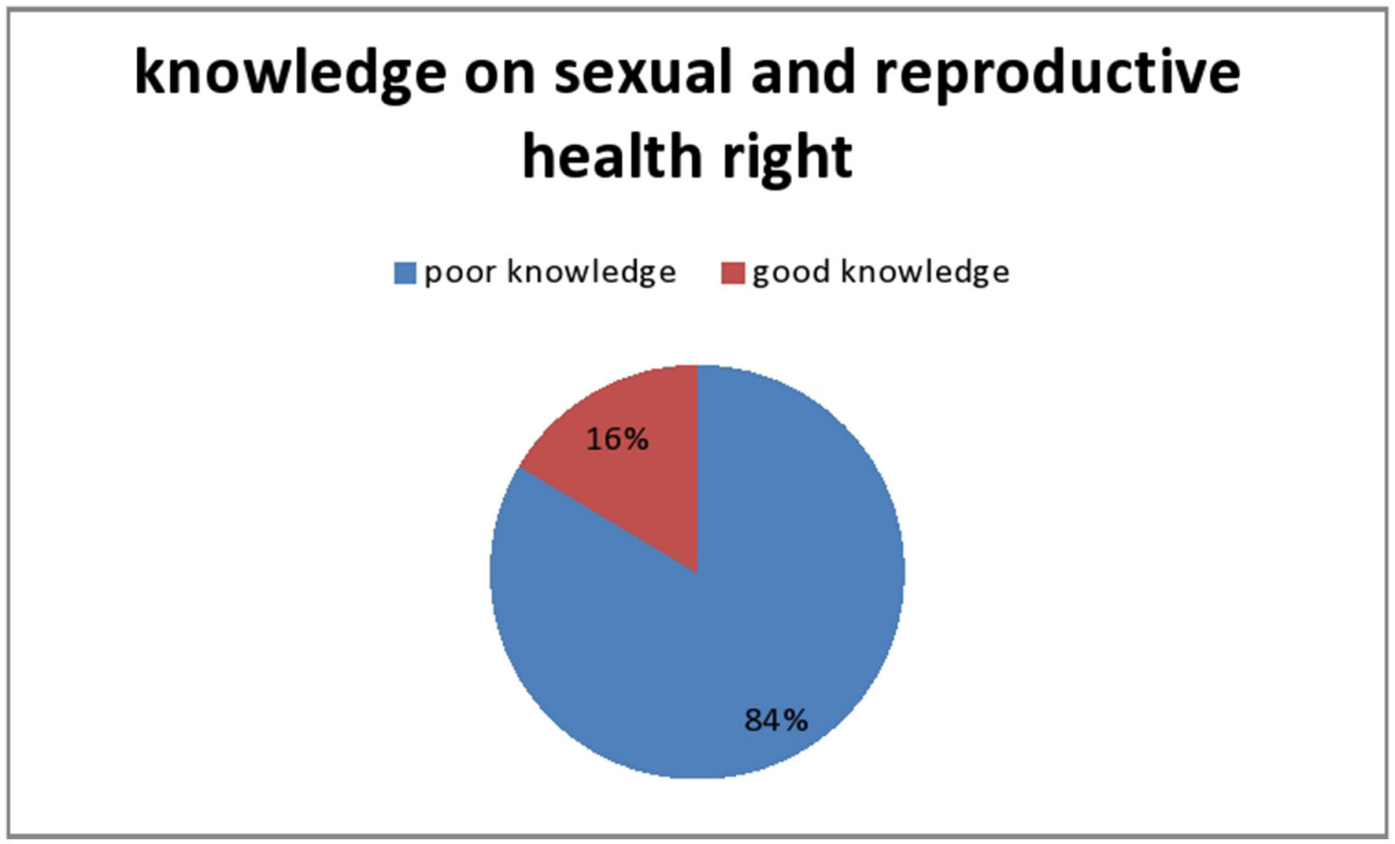
Knowledge on sexual reproductive health rights of regular undergraduate students of University of Aksum, Northwest Ethiopia, 2019 G.C.

Of all the respondents, 330 (79.9%) said that it is important to discuss parenting issues with SRH, with one third (29.1%) having never spoken to anyone in their lives. About 119 (36.5%) people chat with their friends. Participants were asked 24 questions to evaluate their knowledge of reproductive and sexual rights and were grouped into two groups based on their wording. The response rate was 36%. Less than a quarter (16.4%) of respondents were knowledgeable, but not the highest respondent (83.6%) (Figure 2).

### Factors Affecting Knowledge on Sexual and Reproductive Health Rights

In bivariate analysis sex, residence, and religion are the variables. Current sexual partner, previous lecture or education on Rh issues, discussion about Rh with anyone, the problem of unsafe sex, and known information about contraceptive were all associated with knowledge of SRHRs.

In multi-variable analysis; male sex [*AOR*: 1.46, 95% *CI*: (1.09–1.95)]; urban residence [*AOR*: 2.11, 95% *CI*: (1.11–4.91)] previous sexual education or lecture in departments [*AOR:* 1.39, 95% *CI*: (1.02–1.91)] and discussion about Rh issues with anyone [*AOR*; 2.31, 95% *CI* (1.48–3.62)] was significant association with knowledge of SRHRs (Table 2).

**Table 2:** Knowledge of sexual and reproductive health rights about factors associated with participants at Aksum University, Northwest Ethiopia, 2018/2019.

**Variable**	**Category**	**Knowledge**		**COR {*CI*,95%}**	**An OR {*CI*, 95%}**
		**Good%**	**Poor%**		
Sex	Male Female	28 (16.8) 38 (16.2)	139 (83.2) 197 (83.8)	1.04 {1.02, 1.89} **1**	**1.46 {1.09, 1.95}** ^*^ ***1***
Residence	Rural Urban	28 (14.4) 38 (18.3)	166 (85.6) 170 (81.7)	1 1.32 {1.04–1.69}	1 2.11 {1.02, 4.37}^*^
Religion	Orthodox	201 (59.76)	135 (40.24)	1	1
	Muslim	8 (37.78)	15 (62.22)	0.36 (0.22, 0.76)	0.33 (0.18, 0.63)
	Others	22 (51.72)	21 (48.28)	0.70 (0.46, 1.13)	0.66 (0.41, 1.05)
Have a sexual partner currently	Yes No	45 (25.3) 71 (33.0)	133 (74.7) 144 (67.0)	1 1.45 (0.93, 2.26)	1 1.46 (0.89, 2.37)
Previous lecture or education on Rh issues	Yes No	38 (21.4) 30 (12.8)	137 (78.6) 197 (87.2)	1.82 {1.32–1.92} 1	**1.39 {1.02, 1.91}** ^*^ ***1***
Discussion about Rh with anyone	YES NO	28 (23.9) 38 (13.3)	89 (76.1) 247 (86.7)	2.04 {1.04–3.28} 1	**2.31 {1.48, 3.62}** ^*^ ***1***
The problem of unsafe sex	Yes No	15 (57.7) 105 (27.5)	12 (42.3) 270 (72.5)	3.21 (1.59, 8.10) 1	1.68 (0.641, 4.44) 1
Know information about contraceptive	Yes No	106 (27.9) 13 (60.0)	275 (72.1) 8 (40.0)	4.21 (1.54, 9.76) 1	2.26 (0.80, 6.35) 1

* *Found significant at p ≤ 0.05 level of significance*.

## Discussion

Sexual health education largely in response to the HIV epidemic of recent decades, there has been a growing focus on providing young people in sub-Saharan African countries with sexual health education (26). However, national guidelines for sexual education among schools and colleges in Ethiopia have yet to be implemented in the curriculum.

This study aimed to assess the level of knowledge and associated factors on SRHRs in Ethiopia. The goal of sustainable development of reproductive health can't be achieved by not improving access to reproductive health services (27).

The knowledge level of SRHRs was 16.4%. This finding is lower than the knowledge level of SRHR seen in different studies conducted in Ghana (80%) (7), Ikenne (63.2%) (8), Ikorodu (62.3%) and Ikeja (60.3%) (3), Adet Tana Haik college students (59.6%) (9), Gondar University (57.7%) (10), Wolaita Sodo, Ethiopia (54.5%) (2), East Gojjam, Ethiopia (67%) (11), Nigeria (3), Shire town (47.1%) (13), and Harar (31.6%) (12), respectively. This may be due to a lack of standardized sex education (22); culture, and cultural practices that prevent young people from accessing and exercising their SRHRs (28). Besides, in Wolayita Sodo (2) and Adet Tana Haik college (9), the highest proportion of undergraduate students 52 and 68% come from rural areas. Rural living was less likely to be aware of SRH issues (3, 29–32). Therefore, students may have limited information and education on the subject. In the above-mentioned studies conducted in Nigeria, 59 and 62.1% of undergraduate and middle school students, are under two, respectively (3, 33). This discrepancy may be due to the hierarchy of educators. Graduate students may have good general knowledge and experience as they are accessible to a variety of training and courses. Also, students may benefit from the NGO's social and extracurricular activities in the Nigerian public school.

Likewise, according to the logistic regression analysis, significant knowledge opportunities concerning SRHRs have been absorbed among female students (more than 1.5 times). This finding is consistent with the finding in Nigeria (7) because the patriarchal system and unequal gender relations violate woman's rights and limit their participation in society (34). Besides this, the majority (81.5%) of the woman in Nigeria was senior psychologists, who may be aware of their understanding of sexual reproductive health issues. The same finding has been described for human exposure to the media and education, as well as access to public spaces. Social barriers also highlighted the low acceptance differences among women in some societies about their knowledge of condoms and sexuality (35).

According to the study, MA shim's ideology may include a section of social barriers. Women are expected to engage in sexual misconduct, and men are rewarded for their sexual experience.

Students from the urban area are twice as likely as those from rural areas to be educated, which are consistent with other studies in Welayta Sodo University (2) and Gondar University (10). Because students in urban areas have better access to information through youth associations, youth centers, the media, and the environment, whereas most NGOs have limited access to urban areas. However, with the low awareness of the society, which prohibits free and open discussion on reproduction and sexuality, their disgruntled sections may miss out on such opportunities in rural areas.

Our study also revealed students who had discussed the SRH rights with anyone were 2.31 times more likely to be knowledgeable than their counterparts. This study was also consistent with studies conducted at Adet Tana Haik college students (9), Wolaita Sodo University (2), and East Gojjam zone, Ethiopia (11). This can increase the knowledge gained from experience sharing during reproduction and reproductive rights.

Previous Rh-related lecture or education has a positive impact on the knowledge of reproductive and sexual rights. Students who had previously attended lectures or education on Rh issues were 1.43 times more likely to be knowledgeable compared with their counterparts. This is because educational or lecture questions cover a variety of topics related to the subject and students can get accurate information. Studies also show that sex education has increased awareness of sexual reproductive issues (36–41).

The findings of this study are useful in highlighting the level of knowledge and predictors of SRHRs at Aksum University. The findings can help to evaluate sexual and reproductive health strategies, as well as to create benchmark for achieving SDGs and including gender-related goals in health education (25). This would better inform policy debates and decisions at University level in prioritizing interventions, allocate resources, and monitor outcomes. However, those findings need to be cautiously considered during policy debates and priority-setting.

Our finding showed knowledge level the students were very low. This might show less success of sexual and reproductive health policy, strategies and interventions of the Universities on improving knowledge and collaboration with local and international partners.

This study has the following limitations. First, because it is self-reported, under- or over-reporting of information cannot be ruled out. Second, the findings of a cross-sectional study design could not confirm the cause and effect relationship. Third, because a cross-sectional study requires respondents to remember information retrospectively, recoil bias occurs. However, scientific procedure was employed to minimize possible effects. Fourth, weak reliability test of the questionnaire. Only face validity cannot be considered strong evidence for an instrument's validity (42). Additionally, the participants in the study may have misinterpreted certain questions.

## Conclusion

The respondents' knowledge level was found to be very low. Men, residents of urban areas, previous lecture or education on Rh issues, and discussion about sexual issues with anyone else showed a significant positive association with knowledge on SRHRs. Therefore, strengthening students' lectures or education on RH in the curricula of high school, encouraging discussions with parents and anyone might improve the knowledge toward SRH rights.

## Data Availability Statement

The raw data supporting the conclusions of this article will be made available by the authors, without undue reservation.

## Ethics Statement

Ethical clearance was obtained from the Institutional Review Committee (IRC), College of Medicine and Health Sciences, University of Aksum. A Permission letter was received from those faculties and departments. The patients/participants provided their written and verbal informed consent to participate in this study.

## Author Contributions

TEG, EB, and MA designed the study, performed the statistical analysis and data analysis, and drafted the paper. TSG and MH read and approved the final paper. All authors contributed to the article and approved the submitted version.

## Conflict of Interest

The authors declare that the research was conducted in the absence of any commercial or financial relationships that could be construed as a potential conflict of interest.

## Publisher's Note

All claims expressed in this article are solely those of the authors and do not necessarily represent those of their affiliated organizations, or those of the publisher, the editors and the reviewers. Any product that may be evaluated in this article, or claim that may be made by its manufacturer, is not guaranteed or endorsed by the publisher.

## Abbreviations

GBV: Gender-Based Violence
ICPD: International Conference on Population Development
RH: Reproductive Health
RHS: Reproductive Health Services
SDGs: Sustainable Development Goals
SPSS: Statistical Package for Social Science
SRH: Sexual and Reproductive Health
SRHRs: Sexual and Reproductive Health Rights
STIs: Sexually Transmitted Infections.
